# Neuronal immunoproteasome and PFKFB3-forced glycolysis: key players in multiple sclerosis

**DOI:** 10.1038/s41392-025-02372-y

**Published:** 2025-08-29

**Authors:** Claudia Rodríguez-López, Juan P. Bolaños, José J. Lucas

**Affiliations:** 1 Center for Molecular Biology Severo Ochoa (CBM), CSIC/UAM, Madrid, and CIBERNED, Madrid, Spain; 2 Institute of Functional Biology and Genomics, University of Salamanca, CSIC, Institute of Biomedical Research of Salamanca, and CIBERFES, Salamanca, Spain

**Keywords:** Diseases of the nervous system, Neurological disorders

In a recent study published in Cell, Woo et al. ^1^ investigated the role of the immunoproteasome and the metabolic regulator 6-phosphofructo-2-kinase/fructose-2,6-bisphosphatase 3 (PFKFB3) in neurons in the context of multiple sclerosis (MS)-related IFNγ signaling, finding new therapeutic targets for neurodegeneration in MS and possibly other related neuroinflammatory neurodegenerative disorders.

The immunoproteasome is a variant of the constitutive proteasome, where the core catalytic subunits PSMB5 (β5), PSMB6 (β1), and PSMB7 (β2) are replaced by the inducible subunits PSMB8 (β5i), PSMB9 (β1i), and PSMB10 (β2i), which display slightly different hydrolytic profiles. The expression of the inducible subunits increases in most cell types in response to IFNγ, resulting in improved class I antigen processing. However, beyond this immune system function, the immunoproteasome has also been found to play specific regulatory roles, like redox signaling and oxidative stress, among others, in many cell types, including neurons, and the induction of the immunoproteasome in neurons has been implicated in different neurodegenerative diseases, as first reported for Huntington’s disease.^2^

Through snRNA-seq analysis of the experimental autoimmune encephalomyelitis (EAE) mouse model, Woo et al. observed marked transcriptional upregulation of immunoproteasome subunits Psmb8 and Psmb9, with proteomic validation for PSMB8 overexpression. They also observed that IFNγ stimulation of cultured neurons boosted PSMB8 protein levels and downregulated the corresponding constitutive subunit PSMB5, causing altered proteasome activity. Furthermore, neuronal expression of PSMB8 was confirmed in chronic active lesions of people with relapsing MS and in normal-appearing gray matter of people with progressive MS, as well as in various EAE mouse models, therefore correlating neuronal inflammation with proteasome remodeling.

Pharmacological inhibition of PSMB8 with ONX-0914 was confirmed to attenuate disease progression in EAE models, as previously reported by others,^3^ while also conferring protection of neurons against degeneration. Specificity of this observation was corroborated via neuron-specific deletion of Psmb8 in genetically modified mice. Interestingly, these protective effects of PSMB8 suppression occurred without altering immune cell infiltration or antigen presentation, suggesting a cell-autonomous role of PSMB8 in neuronal vulnerability to chronic inflammation.

The authors then moved to demonstrate in experiments performed on long-term neuronal cultures that IFNγ exposure induces replacement of PSMB5 by PSMB8 and that overexpression of PSMB8 (but not PSMB5) leads to neuronal death, which was reverted by co-expression of PSMB5. Furthermore, PSMB8 overexpression escalated ROS levels, reflecting PSMB8–mediated disrupted redox homeostasis along neuronal vulnerability.

**Fig. 1:**
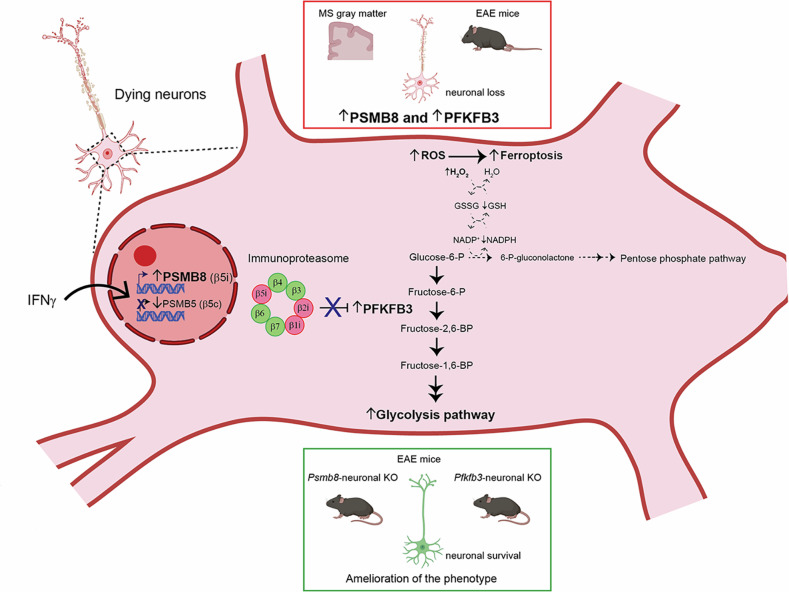
Role of PSMB8 and PFKB3 in neuroinflammation-induced neurodegeneration in multiple sclerosis. Upper: PSMB8 and PFKFB3 are upregulated in neurons of both postmortem MS gray matter and EAE mice. Middle: PSMB8 transcriptional induction by IFNγ in neurons favors the formation of the immunoproteasome, which correlates with increased PFKFB3 levels. Accumulation of PFKFB3 breaks the balance between glycolysis and PPP in detriment of the latter (dotted lines), leading to reduced NADPH synthesis and thus decreased GSH reconstitution. This creates a pro-oxidant environment, which ultimately leads to ferroptosis. Lower: Psmb8 or Pfkfb3 neuronal knock out models improve neuronal pathology in EAE. Some elements of the figure were generated from BioRender

Another consequence of PSMB8-driven impaired proteasome function and subsequent decreased degradation of ubiquitinated proteins was the switch in the metabolism of glucose from the pentose phosphate pathway (PPP) to glycolysis, as the reprogrammed proteasome activity leads to the accumulation of PFKFB3, a key metabolic regulator that promotes glycolysis and that is continuously degraded by the proteasome in neurons.^4^ The suppression of the PPP leads to decreased NADPH availability, thus impeding the regeneration of glutathione, needed for antioxidant balance in neurons. This can ultimately explain the accumulation of redox subproducts such as lipid peroxides, which lead to ferroptosis. The authors demonstrate that these changes, known to occur upon PFKFB3 stabilization,^4^ are directly elicited by IFNγ signaling and subsequent PSMB8 induction through a variety of approaches comprising overexpression, knockout models, and pharmacological experiments in both neuronal cultures and in vivo. They also demonstrate that the CDK5/CDH1 axis, which has been reported to regulate PFKFB3 levels under excitotoxicity, does not play a role in the pathway triggered by IFNγ exposure. Finally, the authors found increased neuronal PFKFB3 staining in *postmortem* MS brains, specifically in cortical areas with chronic active lesions and in normal-appearing gray matter. Interestingly, the amelioration of the EAE course in mice with either *Psmb8* or *Pfkfb3* deletion correlates with reduced neuronal loss and axonal damage. However, demyelination, immune peripheral infiltrates, and microglial activation remained unchanged, indicating that complex cell-type-dependent mechanisms interplay in the pathogenesis of MS-like models of disease. The results of the study are summarized in Fig. 1.

The protective effect of targeting PFKFB3 through pharmacological inhibition or genetic deletion confirms previous observations in a lysosomal neurodegenerative disease model describing how persistent neuronal stabilization of PFKFB3 drives cell death.^5^ In this model, neurotoxicity relies on mitochondrial ROS generation and can be prevented by pharmacological PFKFB3 inhibition, linking defective lysosomal function to metabolic rewiring.^5^ These results also reinforce the broader physiological principle of neurons, unlike astrocytes, being fundamentally ill-equipped for sustained glycolysis and instead relying on astrocyte-derived lactate to fuel oxidative phosphorylation. The astrocyte-neuron lactate shuttle exemplifies this critical metabolic cooperation, wherein glucose is primarily metabolized by astrocytes via glycolysis, and lactate is shuttled to neurons as their preferred oxidative substrate. Although neurons retain the capacity for compartmentalized, short-term glycolysis—for example, in axonal growth cones or during synaptic bursts—such activity is tightly regulated, spatially restricted, and insufficient to meet global energetic or antioxidant demands. Thus, the sustained upregulation of neuronal glycolysis, as seen in the context of immunoproteasome activation and PFKFB3 accumulation, constitutes a pathological deviation from their physiological reliance on astrocytic support. Therefore, the findings by Woo et al. reveal not only a mechanism of neurodegeneration in multiple sclerosis but also a failure of the finely tuned glia-neuron metabolic coupling that sustains neuronal redox homeostasis and viability. Equally, the fact that targeting PFKFB3 through pharmacological inhibition or genetic deletion in neurons during EAE diminishes disease severity and neuronal damage without affecting mitochondrial respiration or immune cell activation confirms PFKFB3 as a key metabolic node mediating neurodegeneration.

In summary, the study by Woo et al. identifies the immunoproteasome subunit PSMB8 as a key driver of inflammation-induced neurodegeneration. In inflamed neurons, IFNγ-induced increase in PSMB8 levels reprograms the proteasome activity, which correlates with the accumulation of PFKFB3. This, in turn, increases glycolysis and suppresses PPP activity, leading to the depletion of antioxidative cofactors such as GSH and sensitizing neurons to ferroptosis. From a therapeutic standpoint, specifically targeting neuronal PSMB8 or PFKFB3 emerges as a new opportunity for therapeutic intervention in MS that deserves future development. Finally, these insights not only deepen our understanding of neurodegeneration in MS but also underscore the importance of preserving neuronal metabolic homeostasis in chronic CNS inflammation.
